# Subjective socioeconomic status and cigarette smoking interact to delay discounting

**DOI:** 10.1186/s40064-015-1361-4

**Published:** 2015-09-28

**Authors:** Keiko Ishii

**Affiliations:** Department of Psychology, Faculty of Letters, Kobe University, Kobe, Japan

**Keywords:** Socioeconomic status, Social class rank, Delay discounting, Cigarette smoking

## Abstract

People generally discount future outcomes, and accordingly accept immediate but smaller gain. This research examined whether this tendency (i.e., delay discounting) is associated with socioeconomic status (SES) and smoking status, and hypothesized that the influence of SES on delay discounting would be moderated by smoking status. Using an Internet survey, 206 participants made choices between receiving hypothetical monetary rewards immediately or with a delay of 1 year. As predicted, the rates of delay discounting were higher as subjective socioeconomic status indicating one’s relative position and standing in a society was lower. Moreover, the tendency was clearer in smokers than in non-smokers, suggesting that cigarette smoking has a moderating effect. In contrast, there was no effect of objective socioeconomic status representing how individuals are able to access valued goods and services.

## Background

People generally discount future outcomes and accordingly accept immediate but smaller gain. The tendency of delay discounting has been explained by some psychological factors such as impulsivity and lack of self-control (for a review, see Green and Myerson 2004; Teuscher and Mitchell 2011). Additionally, the social contexts that individuals live in, such as socioeconomic status (SES), influence delay discounting (e.g., de Wit et al. 2007; Reimers et al. 2009). In this research, I suggest an association between subjective SES, which is indicated by an individual’s judgment of his or her relative rank compared to others in the social hierarchy, and delay discounting. Moreover, I explore the possibility that dependence on nicotine, which is thought to be associated with impulsive behaviors (e.g., Bickel et al. 1999; Ohmura et al. 2005), moderates the association between subjective SES and delay discounting.

Previous findings have shown that higher SES is associated with lower discounting (de Wit et al. 2007; Reimers et al. 2009). These findings usually rely on educational attainment and income as indicators of social status. This reflects an assumption that material resources can be accessed by a combination of education, income, and occupation. Thus, previous findings suggest that people with lower material resources are less likely to take the long-term consequences of their behavior into account.

In addition to these objective indicators, researchers have focused on an individual’s judgment of one’s own rank relative to others as a subjective indicator of social class (see Kraus et al. 2012, for a review). Although objective and subjective indicators are related, they are also independent in that material resources indicated by objective SES factors represent how individuals are able to access valued goods and services, whereas rank indicated by subjective SES characterizes one’s relative position and standing in a society (Kraus et al. 2012). They are indeed positively correlated, but the effect size is moderate (Kraus et al. 2009).

Whereas there is clear evidence of an association between objective SES factors (such as education and income) and delay discounting, little is known about an association between subjective SES and delay discounting. On a related note, Joshi and Fast (2013) demonstrated that power is associated with reduced delay discounting. Given that power is defined as the capacity to control others and outcomes based on the availability of resources (French and Raven 1959) and is rooted in both objective and subjective indicators of SES, it is expected that the association between higher SES and lower discounting would be found even in the case of subjective SES.

Moreover, past research has investigated relationships between substance use, impulsive behaviors, and delay discounting. There is robust evidence that dependence on nicotine is associated with impulsive choices in delay discounting (e.g., Bickel et al. 1999; Ohmura et al. 2005). Thus, smokers are more likely than non-smokers to discount future gain. However, not much is known about the possibility that SES and dependence on nicotine interact to delay discounting. One exception is a study by Jaroni et al. (2004). They found that less educated smokers are more likely to discount future gain compared to more educated smokers. However, because there was no control group (e.g., non-smokers), the study did not address the possibility that the impact of SES indicated by education was affected by dependence on nicotine. Given past research suggesting that a lack of the sense of control is associated with substance use (e.g., Wills et al. 1994) and lower SES (Kraus et al. 2009), discounting behavior would be prevalent in smokers with lower SES.

This research thus examined whether delay discounting is associated with subjective and objective indicators of SES and smoking status, and whether the association between SES and delay discounting is moderated by smoking status. The tendency that lower SES increases delayed discounting would be heightened in smokers rather than non-smokers.

## Method

Two hundred six Japanese adults (125 females and 81 males*, M* age = 44.0 years, *SD* = 12.4) were recruited from a website posted on Micromill, a Japanese web survey company, to participate in the study. Three participants who switched back and forth more than once in response to a series of choices were excluded in the following analyses. Data from the remaining 203 participants are reported here. The participants were compensated with a small amount of money.

The participants were asked to read a hypothetical scenario in which they had just won the lottery and could choose between receiving 25,000 yen (approximately $250) immediately and receiving a different amount of money after 1 year. The amount of money given after 1 year varied from 23,000 to 41,000 yen in increments of 2000 yen in ascending order. The participants were presented with and completed 10 binary choices in total. Following Hardisty and Weber (2009) and Joshi and Fast (2013), this research developed the scenario and used a titration procedure to obtain the indifference point at which future gain was subjectively equivalent to immediate gain. Additionally, following the above-mentioned studies, this research chose the hyperbolic-discounting formula V = A/(1 + kD), where V is the subjective value of a reward, D is the length of the delay, A is the reward amount available at delay D, and k is a free parameter that represents the discount rate. A larger value for k indicates that future outcomes are more discounted and the individual prefers more immediate outcomes. The discount rate was estimated for each participant.

The participants also completed a series of demographic questions, including subjective and objective SES indicators and their smoking behaviors. To measure subjective SES, the participants were presented with a picture of a 10-rung ladder (1: lowest rung, 10: highest rung) and asked to place themselves on the ladder based on where they stood compared to other people in Japan (adopted from Adler et al. 1994). Yearly income was used as an index of objective SES. It was coded into 8 categories ranging from 1 (below 2,000,000 yen) to 8 (above 14,000,000 yen) in increments of 2,000,000 yen. Its demographic information was shown in Table 1. Smoking status was coded as a binary value, based on whether or not the participants were current smokers (0: smoker, 1: non-smoker). In case of current smokers, participants were asked to answer how many cigarettes they usually consume per day. Out of 206 participants, 42 were current smokers (*M*_the number of cigarettes_ = 15.38 cigarettes, *SD* = 9.63). Because the number of cigarettes was not correlated with discount rate in the smokers (*r* = .08, *p* = .61), it was not considered in the following analysis.

**Table 1:** Demographic information of yearly income

Yearly income	%
<2,000,000 yen	44.7
2,000,000–3,999,999 yen	26.2
4,000,000–5,999,999 yen	14.6
6,000,000–7,999,999 yen	10.2
8,000,000–9,999,999 yen	2.4
10,000,000–11,999,999 yen	0.5
12,000,000–13,999,999 yen	1.0
≥ 14,000,000 yen	0.5

## Results

Table 2 shows the mean scores and correlations of SES, smoking status, and discount rate. As in past work (e.g., Kraus et al. 2009), the two indicators of SES were positively correlated (*r* = .26, *p* < .01), but moderately. This suggests that the two indicators were distinct from each other.

**Table 2:** The mean scores of the measures and correlations among them

Measure	M	SD	1	2	3	4
1. Subjective SES	4.59	1.85	–			
2. Objective SES	2.07	1.31	0.26**	–		
3. Smoking status	0.80	0.40	0.15*	−0.13^+^	–	
4. Discount rate	0.20	0.22	−0.14*	0.06	−0.13^+^	–

The ratings of objective SES (yearly income) were based on 8 categories. *1* <2,000,000 yen, *2* 2,000,000–3,999,999 yen, *3* 4,000,000–5,999,999 yen, *4* 6,000,000–7,999,999 yen, *5* 8,000,000–9,999,999 yen, *6* 10,000,000–11,999,999 yen, *7* 12,000,000–13,999,999 yen, *8* ≥ 14,000,000 yen. Regarding smoking status, *0* smoker, *1* non-smoker

** *p* < .01, * *p* < .05, ^+^ *p* < .10

**Fig. 1:**
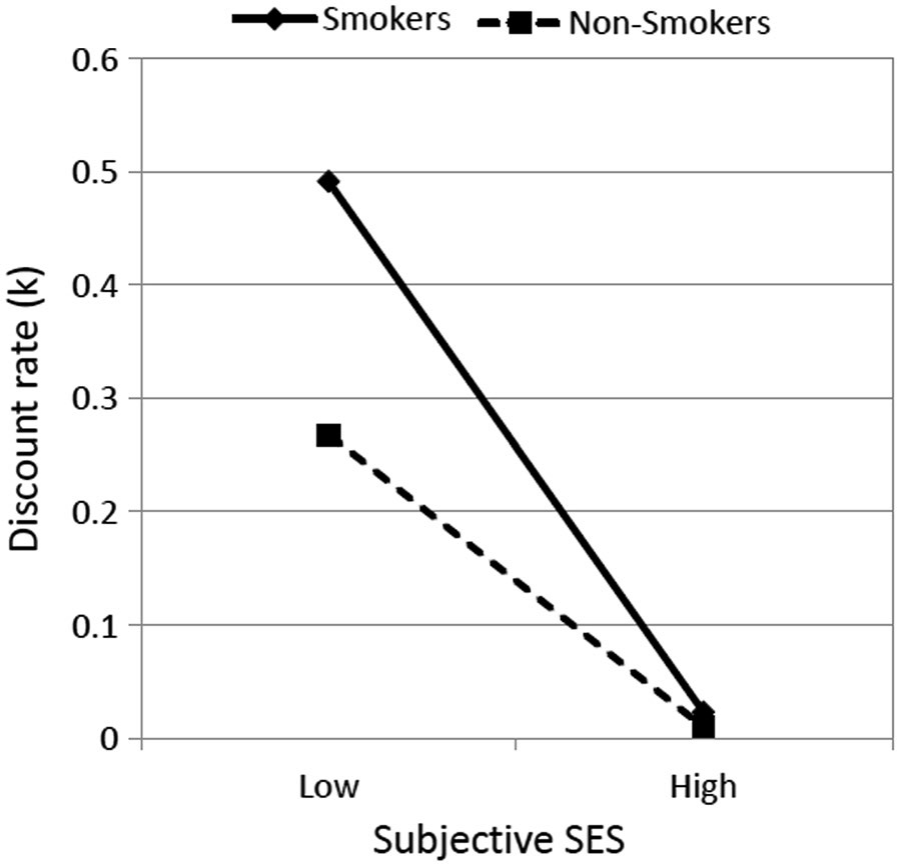
Subjective SES by smoking status interaction predicting discount rate. *Lines* reflect simple slopes for the interactions at low (−1SD) and high (+1SD) levels of subjective SES

A series of multiple regression analyses was conducted. First, subjective and objective SES, smoking status, and demographic variables (gender and age) were entered to predict the mean discount rate (Step 1). Second, the interaction of subjective SES and smoking status was tested (Step 2-A), and the interaction of objective SES and smoking status was tested (Step 2-B). The results of the regression analyses are summarized in Table 3. In Step 2-A, consistent with the hypothesis, the interaction between subjective SES and smoking status proved to be significant, *b* = 0.06, standard error (*SE*) = 0.02, *t*(196) = 3.14, *p* < .01. As illustrated in Fig. 1, regardless of smoking status, individuals with lower subjective SES discounted future outcomes more (smokers: *b* = −0.13, *SE* = 0.04, *t*(196) = −3.52, *p* < .01; non-smokers: *b* = −0.07, *SE* = 0.02, *t*(196) = −3.72, *p* < .001). However, this pattern was attenuated in the non-smoker group. On the other hand, no effect of objective SES was found in the regression analyses, *t*s < 1.27, *p*s > .20.

**Table 3:** The results of a series of multiple regressions predicting discount rate

Predictors	Step 1		Step 2-A		Step2-B	
	*b*	*t* (197)	*b*	*t* (196)	*b*	*t* (196)
Age	0.00	0.69	0.00	0.80	0.00	0.70
Gender	−0.02	−0.61	−0.02	−0.50	−0.02	−0.58
Subjective SES	−0.02	−1.99*	−0.13	−3.52**	−0.02	−1.89^+^
Objective SES	0.01	0.83	0.02	1.26	−0.02	−0.34
Smoking status	−0.04	−1.12	−0.31	−3.33**	−0.08	−1.00
Subj SES × smoking			0.06	3.14**		
Obje SES × smoking					0.02	0.54

** *p* < .01, * *p* < .05, ^+^ *p* < .10

## Discussion

This research demonstrated that lower subjective SES increased delay discounting. This suggests an association between subjective SES and how people wait for larger future rewards. Moreover, to the best of my knowledge, this study offers the first evidence that the slope of subjective SES is steeper in smokers than in non-smokers, and that discounting behavior is apparent in smokers with lower SES. Although the association between smoking behavior and impulsive choice in delay discounting is consistent with previous findings (e.g., Bickel et al. 1999; Ohmura et al. 2005), this study suggests a need to assess the association with an individual’s social class, particularly one indicated by class and hierarchy.

Whereas subjective SES was associated with delay discounting, no effect of objective SES was found. The unexpected findings on objective SES might result from the fact that the present research adopted a categorical measure of yearly income with 8 points in increments of 2,000,000 yen and did not ask for the participants’ exact income. Because the distribution was indeed positively skewed (see Table 1), asking about monthly income and using a categorical measure with a smaller range of money in smaller increments would be more appropriate to detect a relationship between income and delay discounting. Future work testing for samples with a wide range of income is needed.

Past studies have suggested a strong correlation between subjective SES and health indicators such as chronic illness, hypertension, and exercise habits. In addition, a correlation is usually found even if objective SES is controlled for (e.g., Adler et al. 2008). Researchers have assumed that this relationship between SES and health suggests that individuals with lower subjective standing are more vulnerable and have greater responsiveness to stress, reflecting their lack of resources to handle it (e.g., Adler and Snibbe 2003). Moreover, given that social status is associated with serotonergic function fostering impulsive and aggressive behaviors (e.g., Edwards and Kravitz 1997), and that the association leads to poor health behaviors (e.g., Matthews et al. 2010), the current findings demonstrating the relationship between lower subjective SES and delay discounting might align broadly with the findings of past studies on subjective SES and health outcomes in that impulsivity underlies the relationships.

Impulsivity also underlies an association between smoking and delay discounting (Bickel et al. 1999; Ohmura et al. 2005). Nevertheless, the subjective SES and smoking status interaction on delay discounting behavior in this research suggests that smokers and non-smokers do not differ in the behavior when their subjective SES is high. This may result from a sense of control, which is higher in people with higher subjective SES (Kraus et al. 2009), moderates an impulsive choice. However, given that previous studies on the relationship between smoking and discounting have mainly focused on heavy smokers who consume no less than 20 cigarettes per a day (e.g., Bickel et al. 1999), that there is no difference in discounting behavior between non-smokers and mild smokers (Ohmura et al. 2005), and that in the current study only 40 % (17 out of 42) of the smokers are defined as heavy ones, the effect of impulsivity induced by daily nicotine exposure might be weak so that the difference between smokers and non-smokers becomes negligible particularly in people with higher subjective SES.

Although the present research just focused on the effects of demographic factors (SES and smoking status) in delay discounting based on correlations among these factors, it is crucial to explore the underlying mechanisms in future work. The key factors would be impulsivity and sense of control. Future work should examine the role of these factors in the relationships among SES, smoking, and delay discounting and clarify the causal relationships.

There are some shortcomings to the present research. First, it was based on a hypothetical scenario. Although previous studies found no difference between real and hypothetical rewards in terms of delay discounting (e.g., Johnson and Bickel 2002), the effects of SES and smoking might be different if the individuals have to make a choice about real monetary rewards. Second, because this study did not manipulate the participants’ assessment of their relative socioeconomic rank in addition to the cross-sectional nature of the data, the possibility that several factors (e.g., the features of the community where the individuals live) that intervene in the perception of subjective SES may have produced an association with delay discounting cannot be denied. Further investigations that manipulate relative socioeconomic rank (e.g., Piff et al. 2010) will be needed to improve the current findings based on correlations. Finally, this study did not address the effects of SES and smoking on monetary losses, although typically, only monetary gains are examined in most studies. Discount rate for losses tends to be lower than discount rate for gains. Does the difference for outcome effect influence how SES and smoking status interact to delay discounting? In future research, it would be important to see whether the current findings could be extended to the discounting of monetary losses.

## Conclusion

This research reveals an association between relative social class and delay discounting, moderated by smoking status. Future research is needed to focus on impulsivity and the sense of control, which would be linked to these factors, and to seek out an underlying mechanism. Additional insights provided by further investigations based on the current findings would be beneficial for a better understanding of the underlying factors that moderate delay discounting and its consequences.
